# Can Generative Video Address the Clinical Video Data Gap? Evaluation of Synthetic Parkinsonian Hand Motions

**DOI:** 10.64898/2026.10.05.26363719

**Published:** 2026-10-07

**Authors:** Yanbo Feng, Sriharsha Mopidevi, Kaleab Kinfu, Jean Park, Rene Vidal, Kevin B. Johnson

## Abstract

Computer vision approaches to disease motor assessment are limited by clinical video dataset scarcity and distributional imbalance, motivating interest in generative video models as a potential source of training data. We introduce a three-component framework for evaluating the clinical fidelity of synthetic medical motion data, assessing visual fidelity, biomechanical stability, and pathophysiological accuracy, and apply it to videos generated by the text-to-video model Sora 2 depicting Parkinsonian hand-fisting motions across MDS-UPDRS severity levels 0–2 (normal to mild–moderate motor impairment). We further use real-versus-real bootstrapping to compare synthetic-real differences against the natural variability of real clinical videos. Although generated videos appear visually realistic, quantitative analysis shows substantial divergence from real clinical recordings, including weaker severity-dependent patterns of bradykinesia and hypokinesia, less realistic tremor rhythms, and substantial temporal instability. These findings reveal a motion-domain gap between Sora-2-generated and real clinical motor behavior in this Parkinsonian hand-motion setting and demonstrate that visual realism alone is insufficient to establish the clinical utility of synthetic video data for training or evaluating medical computer vision systems.

## Full Text Availability

The license terms selected by the author(s) for this preprint version do not permit archiving in PMC. The full text is available from the preprint server.

